# Novel variants in the *PRDX6 *Gene and the risk of Acute Lung Injury following major trauma

**DOI:** 10.1186/1471-2350-12-77

**Published:** 2011-05-31

**Authors:** Melanie Rushefski, Richard Aplenc, Nuala Meyer, Mingyao Li, Rui Feng, Paul N Lanken, Robert Gallop, Scarlett Bellamy, A Russell Localio, Sheldon I Feinstein, Aron B Fisher, Steven M Albelda, Jason D Christie

**Affiliations:** 1 Division of Pulmonary and Critical Care Medicine, Department of Medicine, University of Pennsylvania School of Medicine, 3600 Spruce Street, Philadelphia, 19104, USA; 2 Department of Biostatistics and Epidemiology, University of Pennsylvania School of Medicine, 423 Guardian Drive, Philadelphia, 19104, USA; 3 Division of Oncology, Children's Hospital of Philadelphia, 34th Street and Civic Center Boulevard, Philadelphia, 19104, USA; 4 Institute for Environmental Medicine, University of Pennsylvania, 3620 Hamilton Walk, Philadelphia, 19104, USA

**Keywords:** Peroxiredoxin, Acute Lung Injury, Oxidant Stress, Genetic Polymorphisms

## Abstract

**Background:**

Peroxiredoxin 6 (*PRDX6*) is involved in redox regulation of the cell and is thought to be protective against oxidant injury. Little is known about genetic variation within the PRDX6 gene and its association with acute lung injury (ALI). In this study we sequenced the *PRDX6 *gene to uncover common variants, and tested association with ALI following major trauma.

**Methods:**

To examine the extent of variation in the *PRDX6 *gene, we performed direct sequencing of the 5' UTR, exons, introns and the 3' UTR in 25 African American cases and controls and 23 European American cases and controls (selected from a cohort study of major trauma), which uncovered 80 SNPs. *In silico *modeling was performed using Patrocles and Transcriptional Element Search System (TESS). Thirty seven novel and tagging SNPs were tested for association with ALI compared with ICU at-risk controls who did not develop ALI in a cohort study of 259 African American and 254 European American subjects that had been admitted to the ICU with major trauma.

**Results:**

Resequencing of critically ill subjects demonstrated 43 novel SNPs not previously reported. Coding regions demonstrated no detectable variation, indicating conservation of the protein. Block haplotype analyses reveal that recombination rates within the gene seem low in both Caucasians and African Americans. Several novel SNPs appeared to have the potential for functional consequence using *in silico *modeling. Chi^2 ^analysis of ALI incidence and genotype showed no significant association between the SNPs in this study and ALI. Haplotype analysis did not reveal any association beyond single SNP analyses.

**Conclusions:**

This study revealed novel SNPs within the *PRDX6 *gene and its 5' and 3' flanking regions via direct sequencing. There was no association found between these SNPs and ALI, possibly due to a low sample size, which was limited to detection of relative risks of 1.93 and above. Future studies may focus on the role of *PRDX6 *genetic variation in other diseases, where oxidative stress is suspected.

## Background

Acute Respiratory Distress Syndrome (ARDS) and Acute Lung Injury (ALI) affect 100,000-150,000 patients each year in the United States alone [1,2]. ALI is an inflammatory syndrome characterized by acute respiratory failure due to non-cardiogenic pulmonary edema and hypoxemia [3]. Oxidant stress caused by reactive oxygen species (ROS) is thought to be a major contributor to the pathogenesis of ALI. ROS can be generated by inflammatory cells or pulmonary endothelium and cause damage to proteins, DNA, and lipids [4].

The risk of developing ALI/ARDS is not uniformly distributed in the critically ill population, suggesting a genetic influence on outcomes [5]. Peroxiredoxins are a superfamily of non-heme and non-selenium peroxidases that are widely distributed throughout all phyla [6]. The Peroxiredoxin 6 gene (*PRDX6*) is located on chromosome 1q24 and is approximately 12 Kb in length, containing 5 exons. The Prdx6 protein encoded by *PRDX6 *is involved in redox regulation of the cell and has been shown in cell and animal models to be protective against oxidative injury [7]. Prdx6 also has phospholipase A_2 _activity and has an important role in lung surfactant metabolism [7]. The protein product, Prdx6, has been shown to affect the cellular level of H_2_O_2 _produced in cells stimulated with platelet-derived growth factor or tumor necrosis factor-α, and modulating signaling induced by those ligands [8], thus indicating that Prdx6 can have an effect on cytokine levels and cell signaling cascades. Recent studies suggest that Prdx6 is only active following heterodimerization with glutathione-S-transferase pi, indicating that there is an important interaction between Prdx6 and GSTpi [6]. Despite these important functions, little is known about genetic variation within the *PRDX6 *gene [9] and its association with ALI.

In order to determine if variation within *PRDX6 *is associated with ALI risk in either the African American (AA) or European American (EA) populations, we performed direct sequencing of the 5' UTR, exons, introns, and the 3' untranslated region (UTR) in 48 subjects (25 African Americans and 23 European Americans) and identified 80 variants, many of which have not been previously reported. Eighteen of the eighty variants, along with 19 tagging SNPs selected using HapMap http://hapmap.ncbi.nlm.nih.gov/, were tested for association with ALI using a custom genotyping platform.

## Methods

### Patient population

Between 1999 and 2006, patients were enrolled in a major trauma cohort study designed to study molecular risks for acute lung injury [10-12]. Participants met the following inclusion criteria: 1) admission to the intensive care unit (ICU) as a result of acute trauma directly from the field or via that hospital's Emergency Department; and 2) have an Injury Severity Score (ISS) ≥ 16 as calculated on the basis of information available during their first 24 hours of hospitalization. The following demographic and clinical variables were collected upon admission to the ICU: age, gender, ISS, blunt mechanism, and acute physiology and chronic health evaluation (APACHE) (Table 1). Exclusion criteria were death or discharge from the ICU within 24 hours of admission, less than 13 years of age, current or past evidence of congestive heart failure (CHF) or recent acute myocardial infarction, severe chronic respiratory disease, morbid obesity, burns on over 30% of the total body surface area, and lung or bone marrow transplant [10].

**Table 1:** Clinical data for individuals enrolled in the study by ancestry

	ALI	No ALI
**African Americans (N = 285)**	(N = 71)	(N = 187)
Age (± SD)	36.7 ± 17.5	31.9 ± 12.4
Gender (% of males )	32	84
ISS (± SD)	25.3 ± 8.9	22.8 ± 6.3
Blunt Mechanism (%) (% of Blunt Trauma)	25	59
APACHE (± SD)	45.4 ± 12.4	40.2 ± 13.5
**European Americans (N = 269)**	(N = 86)	(N = 168)
Age (± SD)	41.8 ± 19.9	39.3 ± 18.5
Gender (% of males )	27	61
ISS (± SD)	26.9 ± 7.5	25.2 ± 7.2
Blunt Mechanism (%)	4	7
APACHE (± SD)	41.9 ± 14.7	35.7 ± 12.8

The definition of ALI was in accordance with the American European Consensus Conference (AECC) [3]. ALI and ARDS were defined as: acute onset; bilateral pulmonary infiltrates on chest X-ray consistent with pulmonary edema; absence of evidence of left atrial hypertension; and poor systemic oxygenation, and a ratio of arterial oxygen (PaO2) to the fraction of inspired oxygen (FiO2) less than or equal to 300 for ALI and 200 for ARDS [3]. All chest x-rays were reviewed independently by 2 trained observers. In our population, greater than 85% of subjects meeting criteria for ALI also met criteria for ARDS.

### Clinical Data and Biosample Collection

Clinical data were collected by trained study nurses using a standardized research case report form designed for the trauma cohort study. Blood for analysis was obtained from residual blood samples in tubes containing ethyledenediaminetetraacetic acid (EDTA) that had been previously drawn for other clinical purposes. Study personnel collected residual samples each day, centrifuged, and separated the buffy coat layers, which were frozen at -80°C [10]. All clinical and biosample collection protocols were approved by the institutional review board (IRB) at the University of Pennsylvania School of Medicine under a waiver of informed consent.

### *PRDX6 *resequencing

Genomic DNA was extracted from whole blood using Qiagen Qiamp DNA Blood Midi Kits (Qiagen USA) and stored in the provided tris-EDTA buffer. DNA from 25 African American and 23 European American subjects selected from the major trauma cohort, with ALI status equally distributed within each group, were selected for sequencing of PCR fragments, providing a power of 99% to detect minor allele frequencies of at least 5% [13]. PCR primers for 4 Kb upstream of the ATG start site, all exons, introns, and 4 Kb of the 3' UTR were designed using PCRoverlap (Children's Hospital of Philadelphia (CHOP) bioinformatics core) to generate amplicons between 600 and 800 bp that overlapped by at least 100 bp (Additional file 1). Following primer optimization, DNA was amplified and sequenced in the forward and reverse direction using a 3730 automated sequencer at the CHOP Nucleic Acid and Protein Core facility (Philadelphia, PA). Sequencher 4.8 (Gene Codes Ann Arbor, MI) was used to facilitate secondary peak calls and to compare the sequence data to the NCBI reference sequence.

### SNP genotyping

Novel variants with a minor allele frequency (MAF) of 0.04 or higher and tagging SNPs, from HapMap http://hapmap.ncbi.nlm.nih.gov/, were validated using the SNPlex genotyping system (Applied Biosystems Inc. Foster City, CA). Tagging SNPs were selected using Tagger's pairwise testing methods described by Bakker and colleagues [14]. Genotyping novel variants not only served to test for association, but allowed us to validate those SNPs in a larger population. Tagging SNPs were also selected to provide better coverage of the haplotype structure of *PRDX6*. SNPlex utilizes an oligonucleotide ligation/PCR assay with universal ZipChute probe detection to perform genotyping of up to 48 SNPs in a single reaction. ZipChute probes were custom designed and detected by capillary electrophoresis using the Applied Biosystems 3130 Analyzer and genotype calls were determined using Gene Mapper 4.0 (Applied Biosystems Foster City, CA).

All genotyping was performed in the University of Pennsylvania's Laboratory for Molecular Epidemiology (LME). Staff was blinded to the disease status and genotyping calls were performed in subsamples by plate. Each plate contained six positive controls to test for concordance. Genotyping calls were performed automatically using the algorithm described by Da La Vega and colleagues [15].

### *In silico *modeling of putative function in SNP sites

We sought to test inferred function *in silico *using transcription factor binding and mRNA binding tools. TESS is a web-based software tool for locating possible transcription factor binding sites in DNA sequences using weight matrix models. It can also be used for browsing information about relevant transcription factors in the TRANSFAC database [16]. All SNPs discovered within the 5' UTR and the first intron were submitted to TESS as 21 base pair long FASTA sequences with the reference allele of the SNP of interest in the 11^th ^position. A second search was performed using the alternative allele in the 11^th ^position. To eliminate any poor matches due to background noise, transcription factors with log-likelihood scores (La) less than 12, were eliminated. TESS results were compared with experimental transcription factor binding site (TFBS) data registered in the University of California Santa Cruz (UCSC) Genome Browser by the Encyclopedia of DNA Elements (ENCODE) consortium [17]. The ENCODE data were filtered by chromosome and position.

A search query was performed for potential miRNA binding sites in the 3' UTR of *PRDX6 *using Patrocles http://www.patrocles.org/. Patrocles is an online database containing DNA sequence polymorphisms that are predicted to interrupt miRNA-mediated gene regulation [18]. The search was performed using "*PRDX6*" as a key word in the target gene id field and miRNA target motifs were defined by Xie *et al. *[19] and Lewis *et al *[20].

### Statistical Analysis of ALI association

259 African American and 254 European American subjects enrolled in the major trauma cohort were used to test for association of novel variants and tagging SNPs with ALI. Association of each *PRDX6 *SNP with ALI was determined separately for European Americans and African Americans using an additive model Chi^2 ^test, with a p-value < 0.0014 for African Americans considered significant. Dominant and recessive inheritance models were also tested using Chi^2 ^analysis. Multivariable analyses of potential confounding were performed using logistic regression methods. Power was calculated using the power for genetic association analyses (PGA) [21]. Using PGA, we estimated that a sample size of 250 subjects per race category would provide 80% power to detect relative risks of 2.26 or greater for SNPs with a prevalence of 0.05 or greater and 1.93 or greater for SNPs with a prevalence of 0.10 or greater, assuming a Bonferroni-corrected alpha = 0.0014 for African Americans, and an incidence of ALI = 0.30 (Additional file 2). These statistical analyses were performed using STATA 11 (STATA Data Corp, College Station, TX). Pairwise linkage disequilibrium was evaluated using Haploview http://www.broadinstitute.org/mpg/haploview. Genotypes with a completion rate of 95% or greater were considered for analysis in Haploview. LD was calculated in terms of r^2 ^values and blocks were defined using the default algorithm using the confidence intervals methods of Gabriel and colleagues [22].

Haplotypes were inferred using the standard expectation maximization algorithm in Haploview [23,24] and the following confidence interval (CI) criteria: CI minima for strong LD: 0.7 - 0.98; upper CI maximum for strong LD: 0.98; fraction of strong LD in informative comparisons ≥ 0.95; and exclude markers with minor allele frequency (MAF) < 0.05. Haplotypes were tested for association with ALI first in a global association test, which performed contingency testing using all haplotypes of an LD block compared to no haplotypes, and then as individual haplotypes versus ALI coded in an additive fashion PLINK [25]. Haplotype multiple testing was addressed by applying permutation tests (10,000 permutations).

## Results

### Identification of novel polymorphisms in *PRDX6*

**Figure 1:**
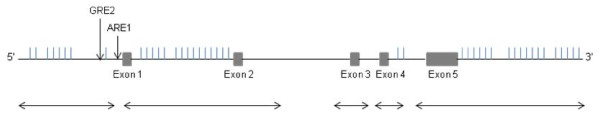
**PRDX6 gene diagram with novel SNP positions**. A schematic of the PRDX6 gene is presented with vertical lines and arrows above the gene indicating novel SNPs and regions of interest, respectively. The horizontal arrows below the schematic are representative of the regions of PRDX6 that were successfully sequenced.

Direct sequencing of the *PRDX6 *gene in 48 subjects revealed 80 genetic variants, none of which were in coding regions (31 in the 3' UTR, 22 in the 5' UTR and 27 intronic) (Additional file 3). The variants identified via direct sequencing were compared with those registered in the NCBI dbSNP database (Build 130) and 43 were found to be novel SNPs (Table 2). Thirty seven were matched with SNPs catalogued in dbSNP (Build 130) and Genewindow http://genewindow.nci.nih.gov/Welcome based on chromosome position (Table 3). Twenty five of the novel SNPs uncovered had a MAF > 0.04 and were submitted to the NCBI to be catalogued and assigned ss numbers in the submitter records section of dbSNP (Table 2). Novel SNPs were also compared with SNPs registered in the 1000 Genomes database. Thirty six out of thirty seven known SNPs overlapped with SNPs registered in 1000 Genomes, but only sixteen out of forty-three novel SNPs identified via our sequencing effort were also registered in 1000 Genomes (Table 4). Several variants were only observed in one individual. As a quality control measure we present the confidence scores for these genotypes in additional file 4. Confidence scores are reported as the percentage of overlap between heterozygote peaks. Previous studies indicated that two transcription factor binding sites, the ARE1 (-357 to -349) [26] and GRE2 (-750 to -738) (A. Fisher, unpublished observations), may play a role in the regulation of *PRDX6*. We were unable to sequence the ARE1 region and portions of the intronic regions due to the GC rich content of the flanking sequence (Figure 1). The GRE2 region was successfully sequenced, but showed no variation.

**Table 2:** Novel SNPs discovered via direct sequencing

Novel SNPs	Region	Chr. Position	SNP	5' Flanking Sequence	3' Flanking Sequence	MAF in EA	MAF in AA	ss# (dbSNP)
PRDX6_171709541	5' UTR	171709541	G/C	CTTCAAGGTTC	ACCCTTATAGC	0.04	0.02	ss217326279

PRDX6_171709910	5' UTR	171709910	G/T	ATGATCATTTTT	GAAATATACAG	0.00	0.10	ss217326288

PRDX6_171710327	5' UTR	171710327	C/T	ACCCTAGCCCC	TGTGCTGGCA	0.04	0.00	ss217326273

PRDX6_171710490	5' UTR	171710490	C/T	TGCACTGCGGA	GCAGGGACCT	0.00	0.06	ss217326283

PRDX6_171710775	5' UTR	171710775	G/C	CTTATGGCTGG	GTGAGACATG	0.00	0.02	

PRDX6_171710821	5' UTR	171710821	C/T	ACTGCACTGAG	TTGTGTAAAGT	0.00	0.10	ss217326292

PRDX6_171711029	5' UTR	171711029	C/T	ACTCAGAGACC	GGGTCCTCCG	0.00	0.02	

PRDX6_171712345	5' UTR	171712345	A/T	ATGGTTCATAA	AGAAAGGGGA	0.87	0.64	ss217326296

PRDX6_171713694	intron	171713694	G/T	TCACTTCCCCG	AGTGCCCAGG	0.00	0.04	ss217326300

PRDX6_171713738	intron	171713738	G/T	CCTCCGTTCTG	TGCTCCCTGG	0.00	0.02	

PRDX6_171713872	intron	171713872	C/T	GCACAAAATGT	TAAAACCACTA	0.00	0.12	ss217326323

PRDX6_171713919	intron	171713919	G/C	AAAGACTTTTTG	AGCCGCCTCC	0.02	0.00	

PRDX6_171714107	intron	171714107	C/T	CCAGGACACGT	TCCCCAACTTT	0.00	0.04	ss217326304

PRDX6_171714984	intron	171714984	C/T	GATCAAAAGTG	TTATCAGGGAG	0.04	0.04	ss217326307

PRDX6_171715019	intron	171715019	A/G	AGGAACACGGT	TATCTGCATTT	0.00	0.10	ss217326318

PRDX6_171715596	intron	171715596	A/G	GGGAGGGAAG	TGAACTGGCTT	0.00	0.02	

PRDX6_171716007	intron	171716007	A/G	AAACCTTGGGA	GTGGCAGCCG	0.00	0.04	ss217326311

PRDX6_171716032	intron	171716032	G/C	TAAGTAGGAAG	TGCCCTTGTCT	0.00	0.02	

PRDX6_171716554	intron	171716554	A/C	AGAAGCCAAGT	AACTTTAATTTT	0.00	0.02	

PRDX6_171716572	intron	171716572	A/T	TCAACTTTAATT	TAAATAGAAGA	0.00	0.02	

PRDX6_171716582	intron	171716582	A/G	TTACATATAAAT	ATAGAAACCTA	0.00	0.04	ss217326315

PRDX6_171716584	intron	171716584	A/T	ACATATAAATAG	AGAAACCTATT	0.00	0.02	

PRDX6_171716603	intron	171716603	A/G	AACCTATTTATT	ATTACATAATTT	0.00	0.02	

PRDX6_171723151	Intron	171723151	C/T	AAAGCTAGCAT	TGGAGAAGAA	0.00	0.02	

PRDX6_171723403	Intron	171723403	C/T	CTTGATTAGTCT	AGCACCTGTAG	0.00	0.02	

PRDX6_171723889	3'UTR	171723889	G/T	AAAACTCAAAT	GGATCTCTGCA	0.00	0.04	

PRDX6_171723918	3'UTR	171723918	A/G	GCTTGTGACCA	GTCATATTTGT	0.00	0.02	

PRDX6_171724000	3'UTR	171724000	G/C	TAACTGTCCTAT	TCCTCTCCTGT	0.00	0.04	ss217326227

PRDX6_171724128	3'UTR	171724128	G/T	TTTTTTTAATAT	TGATCACAGAA	0.00	0.04	ss217326232

PRDX6_171724182	3'UTR	171724182	A/G	CATATTCTTTTA	TCTTGATCACA	0.00	0.04	ss217326236

PRDX6_171724286	3'UTR	171724286	A/T	TTGCTATAAAAA	TTTGTGATAAG	0.00	0.02	

PRDX6_171724949	3'UTR	171724949	C/T	ACTCTACTAATA	CAGGTTTAGAA	0.26	0.00	ss217326270

PRDX6_171725122	3'UTR	171725122	G/T	GGACCTGCTTC	TTGTAGTTTGC	0.00	0.04	ss217326239

PRDX6_171725183	3'UTR	171725183	C/T	GGGATCATCGC	GTCTCATAAGG	0.00	0.04	ss217326242

PRDX6_171725257	3'UTR	171725257	A/T	CCTCCCAAAGG	CATCCAAATAC	0.00	0.04	ss217326247

PRDX6_171725485	3'UTR	171725485	C/T	CCTGCCTCAGC	GAGCAGCTGG	0.00	0.06	ss217326250

PRDX6_171725681	3'UTR	171725681	G/T	ATATTTTTATTG	TAGAATAATGT	0.00	0.02	

PRDX6_171725826	3'UTR	171725826	A/G	TCTGGGGAATG	TTTGAAAGAGA	0.04	0.00	ss217326254

PRDX6_171727146	3'UTR	171727146	G/C	CTGTGATTCCT	TTGTGGTCTTG	0.02	0.00	

PRDX6_171727831	3'UTR	171727831	C/T	ATGCATGGGAT	ATTATCCTCTA	0.04	0.02	ss217326261

PRDX6_171728416	3'UTR	171728416	G/T	CCTCATTAGGG	CTCTTAGCCCT	0.04	0.02	ss217326266

PRDX6_171728455	3'UTR	171728455	G/C	AATCGGGAGGC	TGTTAACAGGT	0.00	0.04	ss217326257

PRDX6_171729049	3'UTR	171729049	C/T	GTTCTTAAACTA	AATAGCATGAG	0.00	0.02	

**Table 3:** Known SNPs discovered via direct sequencing

Knowns SNPs	Region	Chr. Position	SNP	MAF in EA (discovery)	MAF in AA (discovery)	MAF in EA (dbSNP)	MAF in AA (dbSNP)
rs13376447	5' UTR	171709896	A/G	0.00	0.06	0.01	0.09

rs10753081	5' UTR	171710154	C/T	0.39	0.30	0.73	0.21

rs34282688	5' UTR	171710819	C/T	0.07	0.00	NA	NA

rs9425722	5' UTR	171711268	C/T	1.02	0.70	0.04	0.24

rs35152701	5' UTR	171711269	A/G	0.02	0.00	0.00	0.00

rs12739142	5' UTR	171711278	A/G	0.04	0.00	0.02	0.04

rs4354572	5' UTR	171711459	C/T	1.02	0.66	0.00	0.04

rs34619706	5' UTR	171711670	A/G	0.09	0.02	0.07	0.04

rs4382766	5' UTR	171711699	C/T	0.54	0.28	0.30	0.50

rs13376392	5' UTR	171711701	C/T	0.00	0.06	0.00	0.04

rs11576174	5' UTR	171712301	G/T	0.04	0.00	0.15	0.00

rs34977864	5' UTR	171712466	G/T	0.00	0.06	0.00	0.10

rs35441546	5' UTR	171712652	C/T	0.00	0.06	0.00	0.03

rs35133735	5' UTR	171712738	C/T	0.00	0.06	0.00	0.00

rs6671141	intron	171713557	G/T	0.26	0.26	0.24	0.30

rs35749242	intron	171714171	A/G	0.00	0.12	0.00	0.03

rs35918328	intron	171714199	A/G	0.00	0.02	0.00	0.10

rs35899698	intron	171714279	C/T	0.00	0.10	0.00	0.00

rs33942654	intron	171714884	A/G	0.26	0.18	0.23	0.33

rs35679908	intron	171715013	A/G	0.00	0.02	0.00	0.00

rs9425723	intron	171715118	A/G	0.33	0.62	0.23	0.63

rs9425724	intron	171715123	A/G	0.33	0.54	0.23	0.53

rs7540065	intron	171715659	A/G	0.33	0.56	0.23	0.53

rs35244306	intron	171716247	C/T	0.00	0.14	0.00	0.07

rs4611	3'UTR	171723640	C/T	0.33	0.50	0.77	0.47

rs3833536	3'UTR	171724172	C/-	0.00	0.00	0.05	0.04

rs7314	3'UTR	171724222	A/G	0.20	0.50	0.23	0.52

rs36005931	3'UTR	171724224	A/G	0.00	0.04	0.00	0.10

rs2000	3'UTR	171724457	A/G	0.04	0.00	0.03	0.00

rs34129563	3'UTR	171724720	G/C	0.11	0.00	0.02	0.03

rs9425727	3'UTR	171725216	G/C	0.02	0.14	0.00	0.04

rs35358649	3'UTR	171725429	C/T	0.02	0.00	0.00	0.00

rs35547740	3'UTR	171725569	(-/T)	0.00	0.04	0.00	0.07

rs6702835	3'UTR	171725723	A/G	0.33	0.48	0.23	0.54

rs60587131	3'UTR	171726777	G/C	0.00	0.02	0.05	NA

rs57032935	3'UTR	171726836	G/C	0.15	0.22	0.18	0.31

rs6664925	3'UTR	171729033	A/G	0.00	0.18	0.00	0.16

**Table 4:** Comparison of PRDX6 discovery SNPs with 1000 Genomes database

PRDX6 SNPs	1000 Genomes Comparison	PRDX6 SNPs	1000 Genomes Comparison
PRDX6_171709910	Matched	PRDX6_171709541	Not Matched

PRDX6_171713872	Matched	PRDX6_171710327	Not Matched

PRDX6_171714107	Matched	PRDX6_171710490	Not Matched

PRDX6_171723151	Matched	PRDX6_171710775	Not Matched

PRDX6_171723918	Matched	PRDX6_171710821	Not Matched

PRDX6_171724000	Matched	PRDX6_171711029	Not Matched

PRDX6_171724128	Matched	PRDX6_171712345	Not Matched

PRDX6_171724182	Matched	PRDX6_171713694	Not Matched

PRDX6_171725122	Matched	PRDX6_171713738	Not Matched

PRDX6_171725183	Matched	PRDX6_171713919	Not Matched

PRDX6_171725257	Matched	PRDX6_171714984	Not Matched

PRDX6_171725826	Matched	PRDX6_171715019	Not Matched

PRDX6_171727146	Matched	PRDX6_171715596	Not Matched

PRDX6_171727831	Matched	PRDX6_171716007	Not Matched

PRDX6_171728416	Matched	PRDX6_171716032	Not Matched

PRDX6_171728455	Matched	PRDX6_171716554	Not Matched

rs10753081	Matched	PRDX6_171716572	Not Matched

rs11576174	Matched	PRDX6_171716582	Not Matched

rs12739142	Matched	PRDX6_171716584	Not Matched

rs13376392	Matched	PRDX6_171716603	Not Matched

rs13376447	Matched	PRDX6_171723403	Not Matched

rs2000	Matched	PRDX6_171723889	Not Matched

rs33942654	Matched	PRDX6_171724286	Not Matched

rs34129563	Matched	PRDX6_171724949	Not Matched

rs34282688	Matched	PRDX6_171725485	Not Matched

rs34619706	Matched	PRDX6_171725681	Not Matched

rs34977864	Matched	PRDX6_171729049	Not Matched

rs35133735	Matched	rs3833536	Not Matched

rs35152701	Matched		

rs35244306	Matched		

rs35358649	Matched		

rs35441546	Matched		

rs35547740	Matched		

rs35679908	Matched		

rs35749242	Matched		

rs35899698	Matched		

rs35918328	Matched		

rs36005931	Matched		

rs4354572	Matched		

rs4382766	Matched		

rs4611	Matched		

rs57032935	Matched		

rs60587131	Matched		

rs6664925	Matched		

rs6671141	Matched		

rs6702835	Matched		

rs7314	Matched		

rs7540065	Matched		

rs9425722	Matched		

rs9425723	Matched		

rs9425724	Matched		

rs9425727	Matched		

### *In Silico *function of novel SNPs in *PRDX6*

The TESS results showed several potential transcription factor binding motifs in both the reference and alternative sequence. The reference and alternative sequences were submitted as independent queries and transcription factors were returned for 19 positions in the reference sequence and 21 positions in the alternative sequence (Table 5). Twenty seven out of twenty nine sequences submitted were shown to create, abolish, or change a transcription factor binding site. Fourteen of these SNPs were novel. Comparison of the transcription factors returned from the TESS query with the data from ENCODE showed that only 3 of these putative transcription factor binding sites have been tested by the ENCODE consortium, SP1, GATA-1, and c-Myc. ENCODE data for SP1, GATA-1, and c-Myc revealed that there is no evidence of binding affinity with the sequence results from the PRDX6 gene when filtered for *PRDX6 *and A549 cells.

**Table 5:** Potential transcription factor binding sites within the PRDX6 gene

SNP	Location	Base Change	Reference Sequence	Alternative Sequence
PRDX6_171709541	5' UTR	G/C	n/a	Sp1, CACCC binding factor, PuF

rs13376447	5' UTR	A/G	GATA-1	GATA-1

PRDX6_171709910	5' UTR	G/T	n/a	IL-6 RE-BP

rs10753081	5' UTR	C/T	GATA-3	GATA-1

PRDX6_171710327	5' UTR	C/T	C/EBPbeta	C/EBPbeta

PRDX6_171710775	5' UTR	G/C	c-Myb	n/a

rs9425722	5' UTR	C/T	n/a	c-Myc

rs35152701	5' UTR	A/G	n/a	TMF, TBP, TFIID

rs12739142	5' UTR	A/G	IPF1, Isl-1, IPF1	IPF1

rs4354572	5' UTR	C/T	C/EBPbeta	AFP1, ATBF1-B

rs11576174	5' UTR	G/T	H4TF-1	ETF, TMF, TFIID, TBP

PRDX6_171712345	5' UTR	A/T	n/a	SRY, TCF-4E

PRDX6_171712345	5' UTR	A/T	n/a	SRY

rs35441546	5' UTR	C/T	Sp1, ETF	CACCC-binding factor, Sp1

rs35133735	5' UTR	C/T	n/a	CACCC-binding factor, Sp1

rs6671141	intron	G/T	GR alpha, PR, PR A, GR beta	n/a

PRDX6_171713738	intron	G/T	n/a	TEF-1

PRDX6_171713872	intron	C/T	n/a	H4TF-1

rs35918328	intron	A/G	IPF1	n/a

rs33942654	intron	A/G	c-Myb	n/a

PRDX6_171714984	intron	C/T	TBP	GATA-1

PRDX6_171715019	intron	A/G	GATA-3	GATA-1

rs9425723	intron	A/G	TBP, TFIID	AP-1, c-Jun

rs9425724	intron	A/G	USF1	AP-1, AP-4, CCK-1a, c-Myc, CREB, Max1, USF1

PRDX6_171715596	intron	A/G	c-Myb, c-Ets-2	n/a

PRDX6_171716007	intron	A/G	n/a	Sp1, C/EBPbeta, CACCC-binding factor

PRDX6_171716572	intron	A/T	TMF, TFIID, ETF, TBP	n/a

PRDX6_171716582	intron	A/G	GATA-1, GATA-3	n/a

PRDX6_171716584	intron	A/T	GATA-1, GATA-3	SRY

*Transcription factor binding site modeling was performed using TESS.

A Patrocles miRNA database search for *PRDX6 *revealed eight SNPs in the 3' UTR of *PRDX6 *as potential miRNA binding sites (Table 6). Of the eight SNPs returned from the search query, three matched SNPs from this study (rs4611, rs36005931, and rs2000). rs4611 and rs36005931 are located within octamers that have been conserved among several species, but do not correspond to a known miRNA. The G allele of rs2000 is part of an octamer capable of binding miR-942. A literature search for miR-942 returned only sequence data, with no known function to date.

**Table 6:** PRDX6 SNPs thought to be miRNA target sites

SNP ID	Chromosome Position	Base Change	Ancestral	Derived	miRNA
rs4611	171723640	T/C	TTGGTGCT		
				CTGGTGCT	

rs15268	171723695	C/A		AGCAATTA	hsa-miR-302f
				ATTACATA	hsa-miR-380

rs3211528	171724201	G/A	CTGGGGGA		hsa-miR-361-3p

rs36005931	171724224	A/G		GTGCCTTC	
				TGTGCCTT	

rs35820016	171724277	T/A	TTTTGCTA		hsa-miR-548p
				TTGCAATA	

rs1804053	171724351	G/A		ATGTAGCA	hsa-miR-221
					hsa-miR-222

rs11544001	171724433	A/G		GTGCATGA	
				TGCATGAA	
				TGTGCATG	

rs2000	171724457	G/A	AGAGAAGA		hsa-miR-942

*miRNA target site modeling was performed using Patrocles.

### Association of *PRDX6 *with ALI

The trauma cohort described in Table 1 was genotyped for 37 *PRDX6 *SNPs using SNPlex. All SNPs were tested for Hardy-Weinberg equilibrium (Additional file 5). Chi^2 ^analysis of incidence of ALI compared to genotype using an additive model showed no significant association between any of the SNPs in this study and ALI (Table 7). Dominant and recessive models failed to demonstrate an association between our SNPs of interest and ALI (Additional file 6). The genotype concordance rate based on assay positive controls was 100% and the frequency of missing genotypes is presented in Table 7. Logistic regression analysis after adjustment for age and ISS showed no association between ALI and our SNPs (Table 8).

**Table 7:** Association of 37 PRDX6 genotypes and risk of ALI using and additive model in a population of African and European Americans with major trauma

		European Americans				African Americans			
**SNP**	**Source**	**MAF (ALI)**	**MAF (non-ALI)**	**Missing Genotype Frequency**	**p value**	**MAF (ALI)**	**MAF (non-ALI)**	**Missing Genotype Frequency**	**p value**

hCV1948447	Tagging	0.22	0.20	0.016	0.538	0.06	0.05	0.019	0.507

hCV25599136	Tagging	0.00	0.00	0.161	NA	0.03	0.03	0.230	0.842

hCV25599144	Tagging	0.00	0.01	0.399	0.999	0.00	0.00	0.296	NA

hCV9040425	Tagging	0.27	0.25	0.021	0.739	0.38	0.35	0.019	0.725

hCV9040434	Tagging	0.25	0.24	0.407	0.838	0.31	0.29	0.307	0.537

Position23855054	Sequencing	0.00	0.00	0.012	NA	0.09	0.08	0.019	0.535

Position23855203	Sequencing	0.00	0.00	0.679	NA	0.10	0.17	0.662	0.268

Position23859396	Sequencing	0.00	0.00	0.012	NA	0.04	0.02	0.019	0.302

Position23863249	Sequencing	0.00	0.00	0.012	NA	0.03	0.03	0.023	0.577

PRDX6_171709910	Sequencing	0.03	0.01	0.436	0.192	0.04	0.06	0.494	0.505

PRDX6_171711459	Sequencing	0.00	0.00	0.021	NA	0.11	0.18	0.043	0.088

PRDX6_171713872	Sequencing	0.00	0.01	0.037	0.999	0.08	0.05	0.070	0.331

PRDX6_171715019	Sequencing	0.00	0.00	0.029	0.999	0.05	0.05	0.058	0.879

PRDX6_171724949	Sequencing	0.22	0.19	0.029	0.455	0.07	0.05	0.043	0.481

rs10753081	Sequencing	0.34	0.29	0.008	0.246	0.37	0.34	0.000	0.676

rs2000	Tagging	0.05	0.05	0.045	0.966	0.02	0.02	0.031	0.793

rs33942654	Sequencing	0.23	0.20	0.021	0.321	0.34	0.29	0.054	0.138

rs34129563	Sequencing	0.07	0.03	0.021	0.080	0.01	0.01	0.039	0.559

rs34619706	Sequencing	0.08	0.08	0.037	0.900	0.03	0.01	0.047	0.287

rs35244306	Sequencing	0.00	0.01	0.021	0.999	0.11	0.08	0.039	0.517

rs35749242	Sequencing	0.00	0.01	0.021	0.999	0.08	0.06	0.039	0.516

rs4354572	Sequencing	0.00	0.00	0.354	NA	0.11	0.17	0.358	0.251

rs4382766	Sequencing	0.33	0.29	0.025	0.312	0.35	0.34	0.047	0.830

rs4916362	Tagging	0.34	0.29	0.021	0.308	0.35	0.32	0.019	0.670

rs57032935	Sequencing	0.23	0.19	0.037	0.296	0.34	0.29	0.082	0.131

rs6671141	Sequencing	0.23	0.19	0.210	0.421	0.38	0.33	0.163	0.170

rs6699179	Tagging	0.00	0.00	0.214	NA	0.01	0.00	0.171	0.999

rs6702828	Tagging	0.00	0.00	0.012	NA	0.01	0.00	0.027	0.778

rs6702835	Sequencing	0.27	0.25	0.008	0.606	0.42	0.37	0.000	0.493

rs7314	Sequencing	0.27	0.25	0.045	0.666	0.34	0.30	0.078	0.571

rs7367963	Tagging	0.34	0.29	0.029	0.284	0.35	0.31	0.019	0.574

rs7521536	Tagging	0.24	0.20	0.025	0.360	0.32	0.29	0.043	0.320

rs7529377	Tagging	0.24	0.20	0.016	0.331	0.28	0.26	0.027	0.321

rs7540065	Sequencing	0.27	0.25	0.021	0.726	0.37	0.35	0.039	0.997

rs912767	Tagging	0.24	0.20	0.016	0.331	0.29	0.26	0.027	0.240

rs9425722	Sequencing	0.00	0.00	0.342	NA	0.08	0.17	0.335	0.048

rs9425725	Tagging	0.00	0.00	0.012	NA	0.13	0.20	0.027	0.091

**Table 8:** Multivariate analysis adjusted for age and injury severity score

	African Americans						European Americans					
**SNP**	**ORtrend**	**Ptrend**	**ORdom**	**Pdom**	**ORrec**	**Prec**	**ORtrend**	**Ptrend**	**ORdom**	**Pdom**	**ORrec**	**Prec**

hCV1948447	1.33	0.507	1.19	0.705	3.87E+09	0.999	1.16	0.538	1.06	0.840	2.41	0.204

hCV25599136	1.15	0.842	1.15	0.842	NA	NA	NA	NA	NA	NA	NA	NA

hCV25599144	NA	NA	NA	NA	NA	NA	4.19E-10	0.999	4.19E-10	0.999	NA	NA

hCV9040425	1.08	0.725	1.08	0.781	1.13	0.760	1.08	0.739	1.04	0.885	1.32	0.593

hCV9040434	1.17	0.537	1.30	0.442	1.04	0.947	1.06	0.838	0.94	0.871	2.05	0.365

Position23855054	1.25	0.535	1.39	0.398	2.07E-09	0.999	NA	NA	NA	NA	NA	NA

Position23855203	0.56	0.268	0.60	0.374	1.52E-09	0.999	NA	NA	NA	NA	NA	NA

Position23859396	1.73	0.302	2.17	0.190	1.70E-09	0.999	NA	NA	NA	NA	NA	NA

Position23863249	1.36	0.577	1.61	0.431	1.70E-09	0.999	NA	NA	NA	NA	NA	NA

PRDX6_171709910	0.66	0.505	0.72	0.636	1.74E-09	0.999	5.13	0.192	5.13	0.192	NA	NA

PRDX6_171711459	0.58	0.088	0.61	0.146	2.02E-09	0.998	NA	NA	NA	NA	NA	NA

PRDX6_171713872	1.49	0.331	1.69	0.229	1.06E-09	0.999	1.28E-09	0.999	1.28E-09	0.999	NA	NA

PRDX6_171715019	0.93	0.879	1.01	0.989	1.07E-09	0.999	1.77E-09	0.999	1.77E-09	0.999	NA	NA

PRDX6_171724949	1.35	0.481	1.21	0.681	4.18E+09	0.999	1.20	0.455	1.10	0.740	2.50	0.186

rs10753081	1.09	0.676	1.19	0.543	0.98	0.970	1.28	0.246	1.34	0.288	1.44	0.439

rs2000	1.21	0.793	1.21	0.793	NA	NA	0.98	0.966	1.25	0.661	1.30E-09	0.999

rs33942654	1.39	0.138	1.78	0.057	1.05	0.923	1.27	0.321	1.14	0.640	2.96	0.103

rs34129563	1.73	0.559	1.73	0.559	NA	NA	2.16	0.080	2.09	0.115	3.42E+09	0.999

rs34619706	2.13	0.287	2.13	0.287	NA	NA	0.96	0.900	0.89	0.773	2.32	0.554

rs35244306	1.25	0.517	1.41	0.349	1.34E-09	0.999	1.29E-09	0.999	1.29E-09	0.999	NA	NA

rs35749242	1.30	0.516	1.43	0.399	1.09E-09	0.999	1.29E-09	0.999	1.29E-09	0.999	NA	NA

rs4354572	0.65	0.251	0.70	0.386	1.85E-09	0.999	NA	NA	NA	NA	NA	NA

rs4382766	0.96	0.830	0.94	0.838	0.94	0.892	1.24	0.312	1.26	0.409	1.50	0.394

rs4916362	1.09	0.670	1.19	0.551	0.99	0.987	1.24	0.308	1.28	0.381	1.44	0.436

rs57032935	1.40	0.131	1.81	0.053	1.07	0.888	1.29	0.296	1.16	0.600	2.98	0.101

rs6671141	1.37	0.170	1.43	0.256	1.64	0.261	1.24	0.421	1.21	0.549	1.86	0.398

rs6699179	1.14E+09	0.999	1.14E+09	0.999	NA	NA	NA	NA	NA	NA	NA	NA

rs6702828	1.59	0.778	1.59	0.778	NA	NA	NA	NA	NA	NA	NA	NA

rs6702835	1.15	0.493	1.78	0.063	0.61	0.242	1.12	0.606	1.12	0.686	1.28	0.632

rs7314	1.13	0.571	1.26	0.437	1.02	0.963	1.10	0.666	1.07	0.820	1.38	0.542

rs7367963	1.12	0.574	1.13	0.676	1.26	0.589	1.26	0.284	1.30	0.347	1.45	0.432

rs7521536	1.24	0.320	1.47	0.191	1.01	0.977	1.24	0.360	1.11	0.705	2.95	0.104

rs7529377	1.25	0.321	1.31	0.360	1.40	0.506	1.26	0.331	1.14	0.651	2.93	0.106

rs7540065	1.00	0.997	1.03	0.922	0.95	0.896	1.08	0.726	1.04	0.883	1.35	0.570

rs912767	1.30	0.240	1.40	0.250	1.40	0.505	1.26	0.331	1.14	0.651	2.93	0.106

rs9425722	0.44	0.048	0.44	0.065	1.97E-09	0.999	NA	NA	NA	NA	NA	NA

rs9425725	0.60	0.091	0.63	0.154	2.03E-09	0.998	NA	NA	NA	NA	NA	NA

### Haplotype Analysis

Haplotype blocks were created for both African and European Americans using 27 and 28 SNP markers, respectively. Haplotype blocks were created for a region spanning 100.6 kb of chromosome 1. For African Americans, there were 14 SNP markers with genotyping completion rate of less than 95% and were thus excluded from the haplotype analysis. For European Americans, there were 9 SNPs with a genotype completion rate of less than 95% and were excluded from the haplotype analysis.

**Figure 2:**
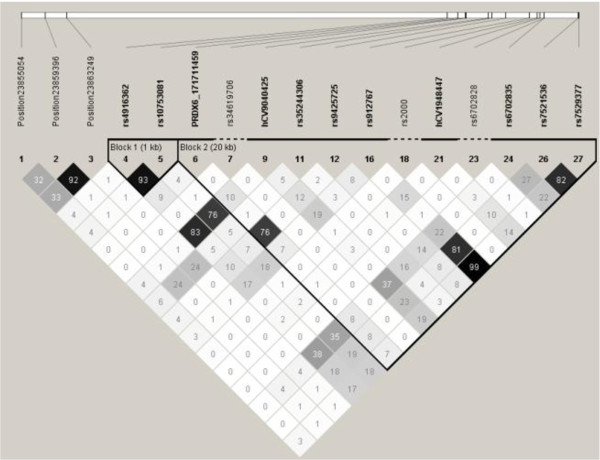
**Haplotype Structure of 17 PRDX6 SNPs in African Americans with r^2 ^Values (N = 259)**.

**Figure 3:**
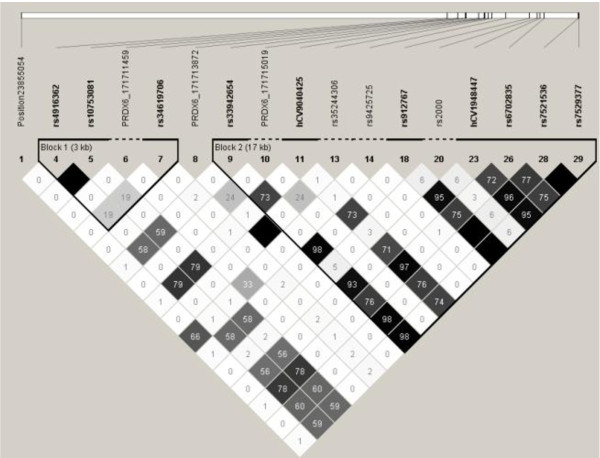
**Haplotype Structure of 17 PRDX6 SNPs in European Americans with r^2 ^Values (N = 254)**.

In African Americans, there were 2 blocks (block1 = rs4916362 and rs10753081; block2 = *PRDX6*_171711459, rs34619706, hCV9040425, rs35244306, rs9425725, rs912767, rs2000, hCV1948447, rs6702828, rs6702835, rs7521536, rs7529377) (Figure 2). In European Americans, there were 2 blocks (block1 = rs4916362, rs10753081, PRDX6_171711459, rs34619706; block2 = rs33942654, PRDX6_171715019, hCV9040425, rs35244306, rs9425725, rs912767, rs2000, hCV1948447, rs6702835, rs7521536, rs7529377) (Figure 3). The haplotype structure of *PRDX6 *appears to be in low LD, in both subgroups. Haplotype analyses of association with ALI did not reveal any significant associations above and single SNP analyses (Table 9).

**Table 9:** Haplotype Analysis among African and European Americans

African Americans				European Americans			
**Block**	**Haplotype**	**Population Frequencies**	**p value**	**Block**	**Haplotype**	**Population Frequencies**	**p value**

Block 1	GT	0.654	0.719	Block 1	ACA	0.690	0.520

	AC	0.330	0.833		GTA	0.230	0.479

	GC	0.016	0.563		GTG	0.080	0.999

Block 2	TGTTACGTG	0.282	0.449	Block 2	GGACGTG	0.734	0.883

	TATTGCACA	0.212	0.386		AAGTACA	0.202	0.634

	CATCACATG	0.153	0.083		GAACATG	0.050	0.297

	TACTACGTG	0.099	0.485				

	TGTTACATG	0.072	0.455				

	TATTACATG	0.055	0.935				

	TATTGTACA	0.051	0.894				

	TATTACACG	0.036	0.969				

	TATCACATG	0.028	0.953				

## Discussion

Prdx6 is a member of the thiol-specific antioxidant protein family and in overexpressing cell and mouse models has been shown to be protective against oxidant stress which null models show sensitivity to oxidants [7,9,27]. Thus, *PRDX6 *is a suitable candidate gene for ALI risk. The extent of genetic variation within *PRDX6 *remains largely unknown, therefore we performed direct sequencing of the *PRDX6 *gene, and identified novel variants for future study. We also tested the newly discovered SNPs and tagging SNPs for association with ALI using our trauma cohort, and did not demonstrate an association with trauma-related ALI.

We identified 43 novel variants among African American and European American subjects with either ALI or control status. None of the 43 SNPs identified were in coding regions which may indicate that the Prdx6 protein is highly conserved across phyla. Approximately 19 kb on chromosome 1 was sequenced in order to achieve adequate coverage of the *PRDX6 *gene and flanking 5' and 3' UTRs. Special attention was given to the GRE2 and ARE1 regions -749 to -737 and -357 to -349, respectively. The ARE1 within the *PRDX6 *promoter was shown to play a role in regulation of transcription and to be inducible under conditions of oxidative stress [26] and the GRE2 may be capable of binding transcription factors under oxidative stress conditions [28]. Due to the GC rich content of the region surrounding the ARE1, we were unable to optimize PCR reaction conditions in a way to prime through the secondary structure. The GRE2 region was sequenced, but no variation was noted. The GC rich region within the *PRDX6 *promoter might warrant further investigation since methylation of DNA cytosine residues are often found in the sequence context CpG. Several new sequencing approaches are emerging that target methylation sites using restriction enzyme treatment followed by sequence by synthesis [29].

In addition to comparing our results with NCBI's dbSNP, we compared our novel and known SNPs with the resequencing data registered in 1000 Genomes. The 1000 Genomes project aims to find most genetic variants with frequencies of at least 1%. Thus far three sequencing projects contribute to the database, low coverage sequencing of 179 individuals from 4 populations, high coverage sequencing of 2 mother-father-child trios, and exon targeting sequencing of 697 individuals from 7 populations [30]. Although 1000 Genomes aims to identify over 95% of variation in any individual, 27 of our novel SNPs and 1 previously recorded SNP are not present in the database, signifying a need for resequencing of extreme phenotypes, such as ALI cases.

Novel and previously recorded SNPs in the 5' UTR and first intron of *PRDX6 *were submitted to TESS to determine their likelihood of being in transcription factor binding sites. We found 19 motifs in the reference sequences that are capable of binding known transcription factors and 21 in the alternative sequence. A comparison between the results of the reference sequence search and the alternative revealed that in most cases, the SNP of interest changes the motif enough to cause a different transcription factor to bind that site or can cause a binding site to disappear and vice versa. After comparison with the ENCODE data, we found that our sequences have not yet been shown to bind the three overlapping transcription factors tested in ENCODE experimentally.

Known SNPs validated in the sequencing effort were compared using a Patrocles search query for miRNA target sites within *PRDX6 *to determine if any of our SNPs were in putative target sites for miRNAs. Three of the eight SNPs returned from the search corresponded with our known SNPs. Only one of the three SNPs was found to have a corresponding known miRNA (miR-942). Some miRNAs are known to control the expression of genes at the posttranscriptional level [31]. However, very limited data are available on miR-942.

We performed an association study for ALI using newly uncovered SNPs and SNPs selected from Hapmap and NCBI's dbSNP and observed no significant association between any of the SNPs in this study and ALI. This lack of association may be due to several causes. First, the detectable effect size is modest because of sample size limitations. We genotyped 513 subjects to test for an association between our selected SNPs and ALI, but this sample size was inadequate to detect relative risks below 1.93 and 1.69 for alleles with MAFs of 0.05 and 0.10, respectively. Second, our analyses were limited to patients with severe trauma. Thus, our study did not evaluate a possible association with other causes of ALI such as sepsis. Finally, it is possible that *PRDX6 *genetic variation may not modify the risk of ALI.

The genotype data were used to construct haplotype blocks to better assess the *PRDX6 *gene structure. Haplotype analysis plays an important role in association studies between genotype and phenotype, since SNPs found to be in strong LD can capture most of the genetic variation across fairly large regions [24]. The haplotype blocks constructed from our genotype data did not show strong linkage disequilibrium using confidence intervals, therefore tagging SNP strategies in future studies should be approached with caution.

Our resequencing data did not show any variation in the coding region of PRDX6. Had nonsynonymous SNPs been discovered, it would have prompted us to investigate whether any of these SNPs had any effect on protein structure, which could cause a loss of function in Prdx6. Since we cannot make a connection between coding region SNPs and conformational changes in the protein, we examined regulatory effects. We found several promoter SNPs that change the sequence of potential TFBSs based on conservation data. We were unable to confirm that these sequences were in fact TFBSs due to the lack of available data. However if any of our promoter SNPs showed a significant association with ALI or another phenotype perhaps using a larger sample size, future studies using promoter constructs could offer more information on upregulation of PRDX6. We also found several SNPs in the 3'UTR. It is possible that one or more of these SNPs is responsible for changing an miRNA binding site, thus repressing protein translation.

Our study has several limitations. One potential limitation of this study is the number of genotype call failures. Ten and nine markers for African Americans and European Americans respectively were eliminated from our analysis since they were under the 95% completion rate cut-off. This high rate of genotype failure was due to difficulties with consistent assay performance rather than DNA quality. If these genotypes had been obtained, it is a possible that an association may have been observed. Also, we did not adjust our results for ancestry informative markers (AIMs). Instead our population was stratified based on skin color, which may not be an adequate proxy for population admixture effects. Another possible limitation is a candidate gene approach that focused on a single gene: *PRDX6*. ALI risk may be considered a complex phenotype, and thus likely is not fully explained by a variation in a single gene [10]. Finally, we only tested for association in patients with ALI from severe trauma. Thus, it is possible that *PRDX6 *may play a role in the initiation or severity of ALI after other insults, including sepsis, or in determining recovery from ALI.

PRDX6 has been shown to play a role not only in ALI, but other diseases as well. A recent studied demonstrated that PRDX6 promotes lung cancer metastasis and invasion via phospholipase A_2 _activity in mice [32]. Another publication reported that PRDX6 transfected breast cancer cells metastasized more readily to the lungs when compared with control cells [26]. It is possible that our novel SNPs may function in lung cancer as well as ALI. The interaction between GSTpi and PRDX6 is another interesting subject for future studies. GSTpi expression is elevated in tumors from a variety of cancers, including lung cancer, compared to normal tissue [33]. Testing gene-gene interactions between PRDX6 and GSTpi would be an interesting future direction both in ALI and other diseases such as cancer.

## Conclusion

In conclusion, this study revealed novel SNPs within the important anti-oxidant *PRDX6 *gene and its 5' and 3' flanking regions via direct sequencing. Several of these variants have putative function and may be useful for future gene association studies. Although there was no association discovered between our novel and tagging SNPs with trauma-related ALI, future studies may focus on the role of *PRDX6 *variation in other at risk groups, as well as other diseases.

## Competing interests

The authors declare that they have no competing interests.

## Authors' contributions

MR carried out the sequencing and genotyping analysis and drafted the manuscript. RA participated in the design of the study, supervision of laboratory assays, and interpretation of data. NM participated in the design of the study, interpretation of data, and manuscript editing. ML performed statistical analyses. RF performed the statistical analyses. PNL participated in the design of the study, collection of data, and manuscript editing. RG performed statistical analyses. SB performed the statistical analyses. ARL performed statistical analyses. SIF participated in the design of the study and interpretation of data. ABF participated in the design of the study, interpretation of data, and manuscript editing. SMA participated in the design of the study, and interpretation of data. JDC participated in the design of the study, data collection, interpretation of the data, manuscript drafting and manuscript editing. All authors read and approved the final manuscript

## Pre-publication history

The pre-publication history for this paper can be accessed here:

http://www.biomedcentral.com/1471-2350/12/77/prepub

## Supplementary Material

Additional file 1 **PCR primers and cycling conditions**. Click here for file

Additional file 2 **Detectable relative risk vs. disease allele frequency**. Click here for file

Additional file 3 **Sequencing variants separated by race and case-control status**. Click here for file

Additional file 4 **Confidence score for sequencing genotypes with only one variant**. Click here for file

Additional file 5 **Hardy-Weinberg equilibrium values**. Click here for file

Additional file 6 **Dominant and recessive models in African Americans and European Americans**. Click here for file
