# Supplementary figures and images for: Novel variants in the PRDX6 Gene and the risk of Acute Lung Injury following major trauma
Source: BMC Med Genet. 2011 May 31;12:77. doi: 10.1186/1471-2350-12-77 (PMC3121666; doi:10.1186/1471-2350-12-77)

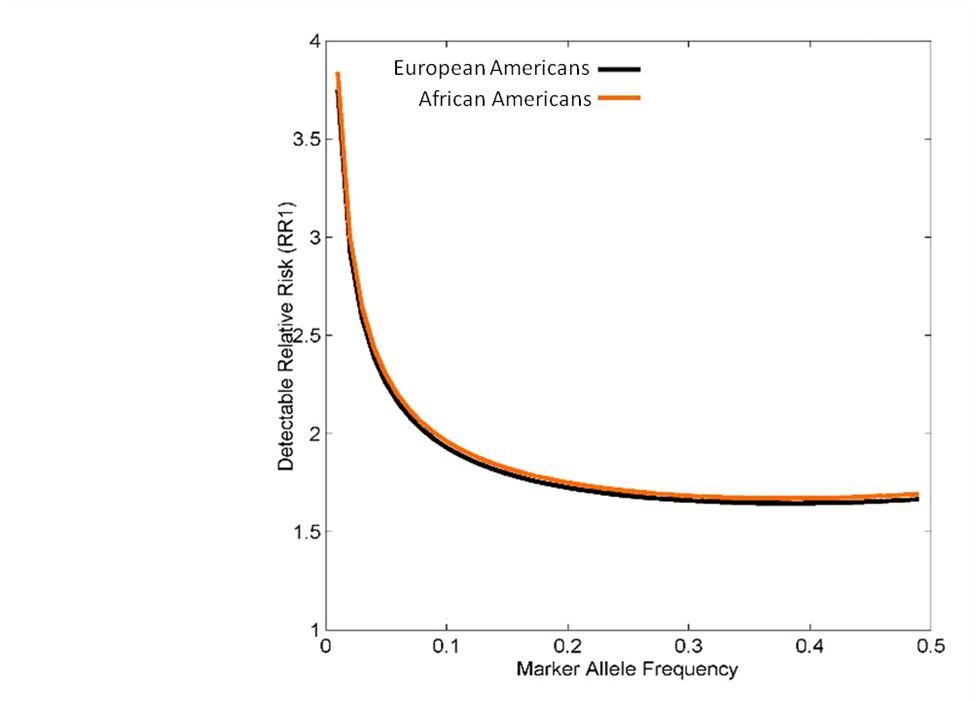

Supplement: Additional file 2 — Detectable relative risk vs. disease allele frequency. [file 1471-2350-12-77-S2.DOC]
